# Rare Metastasis of Prostate Cancer in a Man With Ulcerative Colitis After Creation of an Ileal Pouch

**DOI:** 10.14309/crj.0000000000001552

**Published:** 2024-11-09

**Authors:** Mark Zemanek, Joseph Carter Powers, Katherine Westbrook, Emma Dester, Riley Smith, Taha Qazi

**Affiliations:** 1 Department of Internal Medicine, Cleveland Clinic Foundation, Cleveland, OH; 2 Cleveland Clinic Lerner College of Medicine of Case Western Reserve University, Cleveland, OH; 3 Digestive Disease and Surgery Institute, Cleveland Clinic, Cleveland, OH

**Keywords:** prostate cancer, ulcerative colitis, ileal pouch, anastomosis

## Abstract

Prostate cancer is one of the most common globally diagnosed cancers in men. It most frequently metastasizes to bones, lymph nodes, lungs, or the liver. There are limited data investigating the impact of prostate cancer on patients who have undergone ileal pouch-anal anastomosis. We explore the case and the diagnosis of a 68-year-old man with prostate adenocarcinoma that metastasized to the ileal pouch and ultimately required pouch explant. In addition, we discuss the challenges associated with screening and treating prostate cancer in patients with ileal pouch-anal anastomosis.

## INTRODUCTION

Prostate cancer is a common cancer with an estimated 1.6 million cases diagnosed annually, resulting in approximately 366,000 deaths.^1^ In addition, the incidence of prostate cancer has increased as prostate-specific antigen (PSA) screening has grown.^1^ Men are being diagnosed earlier because of the lead time associated with prostate cancer, which ranges from 3 to 10 years.^1^

Inflammatory bowel disease (IBD) is a group of gastrointestinal inflammatory conditions that include ulcerative colitis (UC) and Crohn's disease. There has been interest between the association of IBD and prostate cancer, given the proximity of the gastrointestinal tract and the prostate.^2^ In a large-scale, prospective, population-based study, there was an association between IBD and prostate cancer with an increased risk of developing prostate cancer in men with UC.^2^

Common sites of prostate cancer metastases include bones, lymphatics, liver, and lungs.^3^ In this report, we present a case of prostate cancer, initially discovered with routine PSA monitoring, that metastasized to an ileal pouch in a patient with a history of UC. Based on this case, we discuss the importance of considering metastatic disease when evaluating patients with otherwise unexplained symptoms. Finally, we explore novel treatment approaches for metastatic prostate cancer and how these may warrant adaptation for patients with an ileal pouch-anal anastomosis (IPAA).

## CASE REPORT

A 68-year-old man with a history of medically refractory UC that had been surgically managed with IPAA creation presented to his primary care physician 5 years after IPAA in the setting of recurrent inflammatory pouch symptoms. During this period, he experienced worsening symptoms of increased bowel movement (BM) frequency and incomplete evacuation, despite having a grossly normal pouch endoscopic evaluation (pouchoscopy). Furthermore, his symptoms were only partially abated with antibiotics and antidiarrheals.

At this time, the patient was found to have an elevated PSA of 14.13 ng/mL, and it remained elevated a month later. Given concern for prostate cancer in a patient with worsening ileal pouch symptoms, he was referred to gastroenterology (GI) and urology. GI planned for an anorectal manometry and X-ray defecography to exclude mechanical sources of pouch dysfunction. The results suggested pelvic floor dysfunction, and he was referred to pelvic floor therapy. Given weight loss, increased stooling, and an elevated PSA, urology was concerned for an underlying malignant process.

As his workup with GI and urology progressed, the patient experienced increased BM frequency (10–15 BM daily) and worsening dyschezia. GI initiated amoxicillin/clavulanate which only minimally improved frequency. Because of persistent symptoms, a repeat pouchoscopy was obtained showing a congested rectal cuff and erythema with granularity in the ileoanal pouch. X-ray defecography was unremarkable. Pathology from the pouch evaluation showed evidence of chronic active enteritis. Despite mild inflammation in the pouch, the results did not fully correlate with the patient's clinical picture. Given the suspected prostate cancer, GI elected to await the prostatectomy for additional management considerations.

The patient underwent a transperineal prostate biopsy and later was scheduled for a radical prostatectomy, which revealed prostatic adenocarcinoma Gleason score 7 (3 + 4), Grade Group 2, with extraprostatic extension. After prostatectomy, the patient's PSA postoperatively decreased to 2.04 ng/mL and he had an initial improvement in symptoms with intensive pelvic floor retraining and intermittent antibiotics. However, he gradually reported increasing BM daily, fecal seepage, and incontinence. He was, therefore, connected with an IBD-specialized dietician who encouraged a low roughage diet. He was also evaluated by a GI psychologist who recommended a trial of amitriptyline and focusing on mindfulness. Anorectal manometry showed improvement in pelvic floor dynamics.

During follow-up with urology 3 months after operation, repeat prostate-specific membrane antigen (PSMA) positron emission computed tomography was negative for metastasis. Radiation oncology discussed the patient's options, which included active surveillance, external beam radiation therapy (EBRT), and androgen deprivation therapy (ADT). A mildly elevated PSA without a known source was noted, but radiotherapy was not recommended with a negative PSMA positron emission computed tomography scan. The patient was referred to medical oncology and ultimately declined ADT and elected for PSA observation.

**Figure 1.**
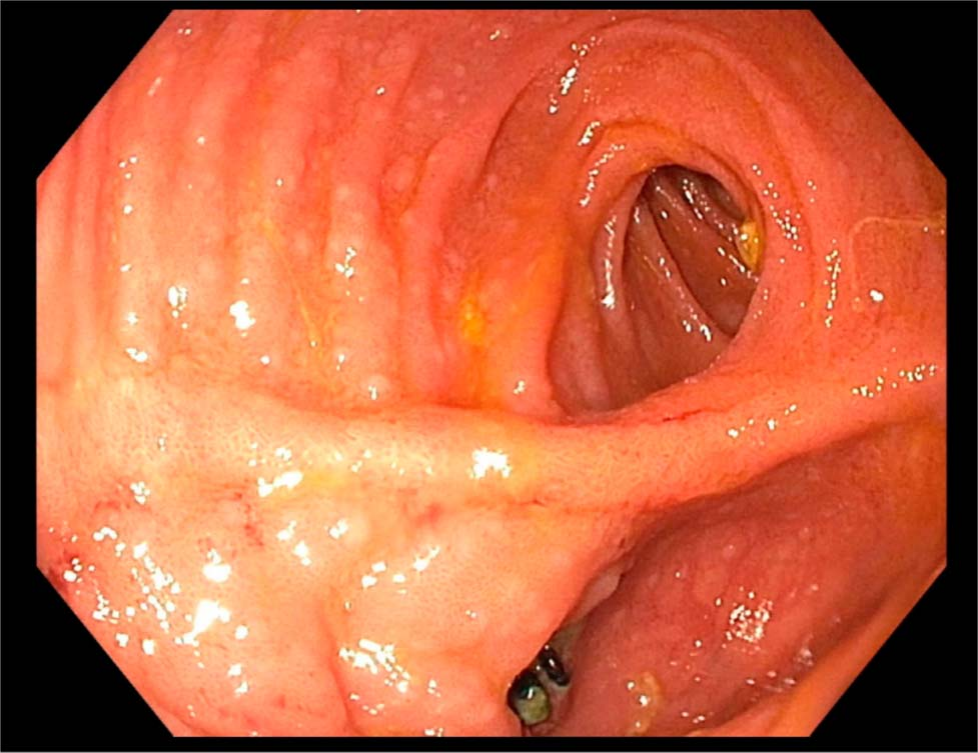
Pouchoscopy with biopsy.

**Figure 2.**
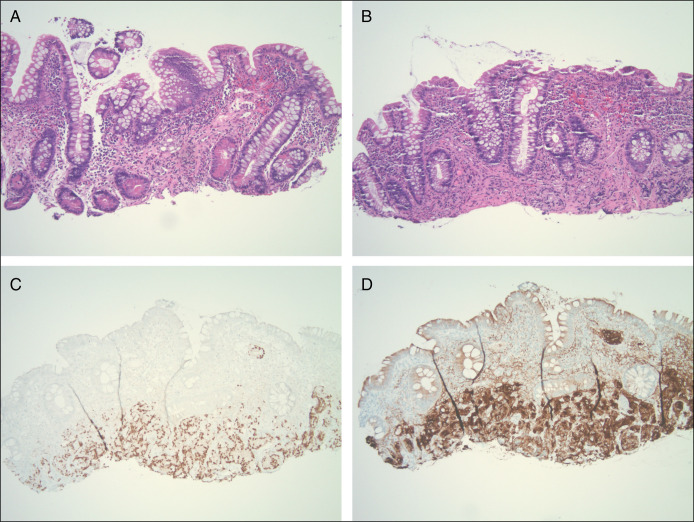
(A, B) Prostate biopsy hematoxylin and eosin. Histologic sections showed atypical glands with foamy mucinous cytoplasm and prominent round nuclei infiltrating the small intestinal mucosa and lymphovascular spaces. (C) NKX3 stain. (D) Prostate-specific membrane antigen stain. Pathology slides: 10x objective, 100x magnification.

Six months after prostatectomy, the patient's PSA continued to trend upward (2.36–3.0). A repeat pouchoscopy showed congestion in the rectal cuff and mild inflammation in the distal posterior pouch that was believed to be related to previous cancer therapy (Figure 1). However, biopsy of this region revealed metastatic prostate cancer. Histologic sections showed atypical glands and prominent round nuclei infiltrating the small intestinal mucosa and lymphovascular spaces (Figure 2). Malignant cells were PSMA and NKXS.1 positive (Figure 2).

**Figure 3.**
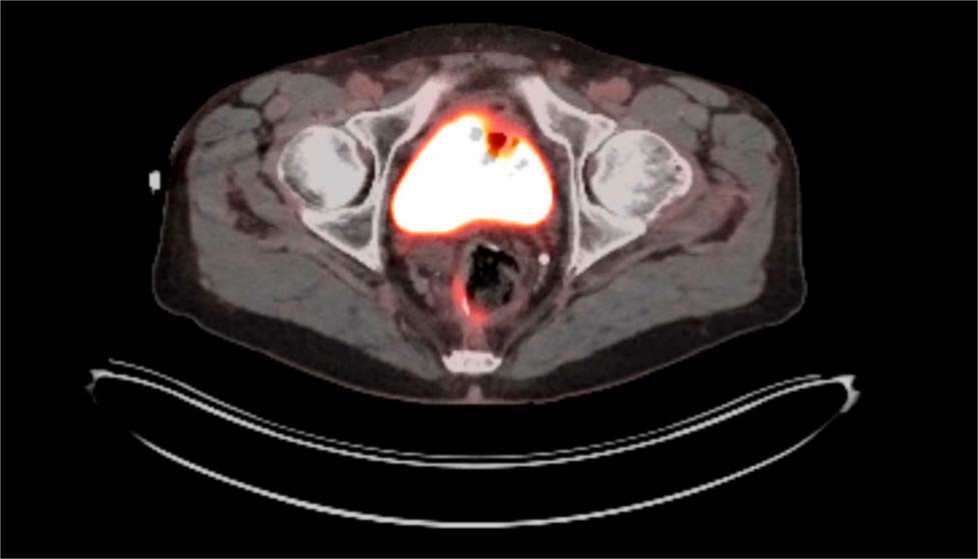
Repeat PSMA positron emission computed tomography imaging showing postsurgical changes from the radical prostatectomy and J-pouch creation with moderate PSMA expression along the right posterolateral margin of the J-pouch that was compatible with metastasis. PSMA, prostate-specific membrane antigen.

**Figure 4.**
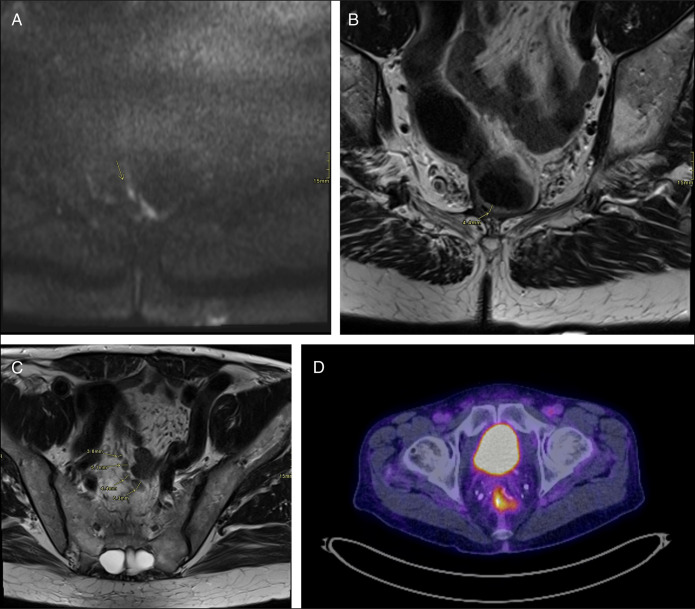
(A, B) Crescent of restricted diffusion along the area of PSMA uptake in the right lateral J-pouch (arrow) and is associated with an area of thickening on the axial T2 image. (C) Prominent mesorecctal nodes (arrows). (D) Relevant PSMA positron emission tomography image. Surgical change of J-pouch with nonspecific uptake along the right and inferior J-pouch wall. PSMA, prostate-specific membrane antigen.

Given these findings, the patient was referred to oncology. Oncology recommended repeat imaging which showed postsurgical changes from the radical prostatectomy and IPAA with moderate PSMA expression along the right posterolateral margin of the ileal pouch, compatible with metastasis (Figure 3). There were no PSMA-expressing lymph nodes or additional metastases identified. The patient was referred to a tumor board, and magnetic resonance imaging confirmed pouch metastases (Figure 4). The patient ultimately elected to pursue pouch explant with a permanent end ileostomy.

## DISCUSSION

This case highlights the importance of extensive investigation into unexplained patient symptoms. Despite evaluation by numerous specialists, an explanation for the patient's increased frequency and discomfort remained elusive. Clinical consideration for metastatic pouch disease must remain on the differential, particularly in patients with previous malignancy.

Once diagnosed, prostate cancer treatment presents another hurdle by requiring a multifaceted approach of surveillance, surgery, radiation, hormone therapy, chemotherapy, and/or immunotherapy. Surveillance includes monitoring PSA and if necessary, further evaluation with biopsy.^4^ Treatment options may include radical prostatectomy and EBRT.

Standard therapy for prostate cancer after radical prostatectomy typically includes radiation and ADT.^5^ However, radiation therapy was not offered to this patient given his IBD history, and he elected to not pursue ADT. There is limited information on the role of ADT use for patients with IPAA, representing a knowledge gap that should be explored further.

In a study of 30 patients with IPAA and prostate cancer, the risk of pouch failure was increased after prostate cancer diagnosis, regardless of whether cancer treatment was pursued.^5^ Specifically, they looked at patients with IPAA with prostate cancer and matched controls that were offered treatment modalities including prostatectomy, brachytherapy, watchful waiting, and hormonal therapy, similar to our patient case.^5^ For patients in the study who retained their pouches, there was not a significant change in pouch function or quality of life.^5^

Beyond gaps in the literature, challenges remain for screening recommendations for patients with IPAA once diagnosed.^6^ No guidelines exist for responding to elevated PSA values in this population and addressing rare metastatic sites.^7^

There remain many challenges for patients who have undergone proctocolectomy with IPAA that require further surgical intervention with radical prostatectomy given concerns of previous pelvic surgery with altered anatomy.^8^ As well, EBRT is a contraindication given the radiosensitivity of small bowel mucosa. Brachytherapy is a safer alternative but has limited oncologic effectiveness.^9^ Furthermore, there is limited knowledge on the impacts of oncologic outcomes in patients with IPAA after prostatectomy.^8^

Given the high incidence of prostate cancer, it is essential to implement monitoring strategies to best capture and treat these patients. This case highlights that consideration of metastatic cancer for refractory ileal pouch symptoms is essential to capture and manage invasive disease.

## DISCLOSURES

Author contributions: M. Zemanek is the primary author who performed literature review on the topic, drafted the case report and helped with draft revisions. J. Powers assisted with literature review, draft preparation and revisions. K. Westbrook, E. Dester, and R. Smith helped assist with draft revisions. Dr. T. Qazi was the primary attending physician who managed this patient and assisted with literature review and draft revisions. M. Zemanek is the article guarantor.

Financial disclosure: JC Powers: The Crohn's and Colitis Foundation Grant Funding through the Student Research Fellowship Award (Grant/Research Support). T. Qazi: AbbVie Bioscience (Advisor or Review Panel Member, Consultant, Grant/Research Support, Speakers Bureau); Celgene/BMS (Advisor or Review Panel Member, Speakers Bureau); Janssen (Speakers Bureau); Pfizer (Advisor or Review Panel Member, Advisory Committee/Board Member); Prometheus Biosciences (Advisor or Review Panel Member, Advisory Committee/Board Member, Consultant).

Informed consent was obtained for this case report.

## References

[1] Pernar CH Ebot EM Wilson KM Mucci LA. The epidemiology of prostate cancer. Cold Spring Harbor Perspect Med. 2018;8(12):a030361. 10.1101/cshperspect.a030361 PMC6280714 29311132

[2] Meyers TJ Weiner AB Graff RE . Association between inflammatory bowel disease and prostate cancer: A large-scale, prospective, population-based study. Int J Cancer. 2020;147(10):2735–42. 32399975 10.1002/ijc.33048 PMC7577830

[3] Vinjamoori AH Jagannathan JP Shinagare AB . Atypical metastases from prostate cancer: 10-year experience at a single institution. AJR Am J Roentgenol. 2012;199(2):367–72. 22826398 10.2214/AJR.11.7533

[4] Chen RC Rumble RB Loblaw DA . Active surveillance for the management of localized prostate cancer (Cancer Care Ontario guideline): American Society of Clinical Oncology Clinical Practice Guideline Endorsement. J Clin Oncol. 2016;34(18):2182–90. 26884580 10.1200/JCO.2015.65.7759

[5] Lian L Ashburn J Remer EM Remzi FH Monga M Shen B. Impact of prostate cancer and its treatment on the outcomes of ileal pouch-anal anastomosis. Inflamm Bowel Dis. 2017;23(12):2147–53. 29135694 10.1097/MIB.0000000000001263

[6] Shen B Angermeier KW Remzi FH Katz S. Screening and diagnosis of prostate cancer in patients with ileal pouch–anal anastomosis: Consensus from an expert panel. Am J Gastroenterol. 2011;106(2):186–9. 21301448 10.1038/ajg.2010.300

[7] Gandaglia G Abdollah F Schiffmann J . Distribution of metastatic sites in patients with prostate cancer: A population-based analysis. Prostate. 2014;74(2):210–6. 24132735 10.1002/pros.22742

[8] Uchino T Lincango E Liska D . S126 pouch urinary fistulae after ileal pouch-anal anastomosis is associated with decreased pouch survival. Am J Gastroenterol. 2022;117(12S):S32–S33.

[9] Pedraza AM Ferguson EL Ramos-Carpinteyro R . Managing prostate cancer after proctocolectomy and ileal pouch-anal anastomosis: Feasibility and outcomes of single-port transvesical robot-assisted radical prostatectomy. World J Urol. 2024;42(1):368. 38832957 10.1007/s00345-024-05051-9 PMC11150298

